# Vis Medicatrix Naturæ and Antistasis

**Published:** 1855-12

**Authors:** John W. Jones

**Affiliations:** Professor of the Principals and Practice of Medicine in the Atlanta Medical College


ATLANTA

### Medical and Surgical Journal.

VOL. I.] DECEMBER, 1855. [No. 4.

### ORIGINAL COMMUNICATIONS.

ARTICLE I.—" *Vis Medicatrix Naturæ and Antistasis.*" By JOHN W. JONES, M. D., Professor of the Principles and Practice of Medicine in the Atlanta Medical College.

The art of printing is no less valuable to the people of the present age than it was to those who lived cotemporary with Guttemburg, Faust and Schœffer, respectively its inventor, promoter and improver; and who jointly published to the world "*adinventionem artificiosam imprimendi ac characterizandi.*" The advantages that have accrued to mankind from the discovery of the western continent had but begun when Christopher Columbus beheld, amidst hopes and fears, for the first time, in the dim distance, the coast of San Salvador. The Elements of Euclid, the Principia of Newton, the Organon of Bacon, and the Copernican system as contrasted with the Ptolemaic, will be as new and perhaps as valuable to future generations as they were to those to whom they were first promulged and expounded. Are we, of the present century, basking—as we certainly are—in the meridian sun-shine of civil and religious liberty, together with all the blessings common to our great and happy republic, to forget the priceless boon bequeathed us by our patriot fathers of '76? Are the motives and the principles which actuated our noble sires of the Revolution to deeds of unparalelled greatness, and which have been so graphically proclaimed in the Declaration of Independence, the Constitution of the United States and the Farewell Address of our great political father, the illustrious Washington, to diminish in interest or value with the mere lapse of time? We hope not. The decalogue is as important to the welfare of the present generation as it was to the journeying Israelites who stood at the trembling base of smoking Sinai when Moses received the tables of stone from the hand of Jehovah. And the Sermon on the Mount is as essential to our salvation as it was to those who listened to the preaching of Him "who spoke as man never spake." Why, then, reasoning *apriori*, should the mouldering hand of time be permitted to obscure the bright and shining lights that were kindled upon the ancient altars of medical science by the genius of those who first worshiped around them; lights which have for centuries illumined the paths of the practitioner of the healing art amidst the dubious and discouraging scenes of his chemical ministrations, and which have enabled him to snatch his desponding and emaciated patients from the gloomy borders of the grave and conduct them to the universally coveted climes of health and longevity; lights which should no longer be kept beneath the *bushel*, but rather be kept trimmed and burning? Therefore, to the Veteran and the Tyro, to each and all, our suggestion is "*alere flammam.*" However much and justly we, as a profession, may boast of the achievements of medical science in the amelioration of human suffering, and in the prolongation of human life, nevertheless, we are compelled to know, and constrained to admit, that until we shall have attained to a still more accurate and comprehensive knowledge of the laws of life, the causes of disease, its diagnosis, and the various processes by which physiological dissolution ensues, as death by anœmia, asthœnia, inanition, apnœa, coma, &c., as well as a more intimate acquaintance with the *modus operandi* with remedial agents, that our ancient and honorable *art* is yet too subject to the humiliating and opprobious epithet of the "uncertain science;" that it is too limited in its resources, too meagre in its claims, too equivocal, if not precarious, in its tendencies and results, and too circumscribed in its benefits to mankind; and that it also falls far short of that glorious exaltation and hygienic era, so long and enthusiastically predicted by its disciples. And yet, while this is the case, we are rejoiced to know, that much that is valuable has been achieved, and would fain hope that its future may develop a still richer contribution.

In this communication, which is only intended as an epitome of our opinions upon the subject that constitues its caption, our main object is, to awaken in the minds of medical readers and practitioners, the spirit of a more general and thorough consideration of this topic than at present seems to exist.

To be learned, is not necessarily to be wise, and although it cannot be denied that a majority of physicians, in this our day, enjoy greater facilities for the acquirement of professional knowledge than those of any former period, yet we are led to question whether that knowledge is *pro rata* as judiciously applied. That our therapeutic agencies are capable of effecting much that is valuable in the counteraction and removal of disease, has certainly been fully and extensively demonstrated.—And while we have no disposition, or intention, to disparage the claims of the materia medica, we can only award to it the place of an auxiliary in the curement of disease, and even that influence is, we believe, ever dependent for salutary results upon the *vis preservatrix naturæ.* On this subject, the gifted and highly distinguished Chapman, of Philadelphia, in the first paragraph of his views upon the *modus operandi* of medicines, says: "Not a little difference of opinion prevails on this very intricate question. The only point in the controversy which seems to be conceded is, that the operation of medicines does not depend on any of the common laws of matter, but on a principle incident to vitality alone." And then, in confirmation of this fact, quotes the established maxim, "*Medicamenta non agunt in cadaver.*"

The practitioner who would successfully combat the various and malignant forms of morbid action, must first familiarize himself with the fundamental principles of life, health and disease. He must consult and, as far as possible, imitate by his therapeutic processes, the operations of nature, and especially must he understand the laws that regulate the normal and abnormal phenomena of the animal economy.

That principle in the animal organism, that has for its object the preservation of our physiological existence, has been termed by the earlier writers the *vis vitic*, and we learn from the medical records of antiquity, that philosophers were induced to believe that a great part of the phenomena peculiar to living bodies did not follow the same course, nor obey the same laws, as the phenomena common to inanimate matter. To the phenomena of animated nature a paticular cause has been assigned which has been variously defined. Hippocrates called it *physis*, or nature. Stahl, in his "*Theoria Medica Vera*," denominated it the *soul*. Aristotle named it "the *moving*, or *generating principle*. Bœrhave, the *impetum faciens*. Van Helmont, *Archœa*. Others, the *vis insita*, *vis vitœ*, *vital force*, &c. This principle, it was believed, was chiefly operative in the maintenance of life and health, unassailed by the causes of morbid action. But a more extended and matured observation of the laws of phenomena of matured observation of the laws and phenomena of life and health, disease and death, suggested the idea that it was also the *conservative* agent by which the encroachments of disease were resisted, and by which, in the main, its expulsion from the system was effected, and hence the origin of the doctrine *Antocrateia*, or "*vis conservatrix vel medicatrix naturœ*." That is, "That healing power in an animate body by which, when diseased, it is enabled to regain its healthy actions" independent of artificial assistance. This great fundamental truth originated with the existence of animated nature, and as a doctrine in medicine it was promulged by the illustrious Hippocrates, to whom has generally been awarded the enviable and honored title of "Father of Medicine," and who laid the foundation of medical science. This master spirit has been reckoned the eighteenth lineal descendant from Æsculapius, the profession of medicine having been transmitted hereditarily from the latter to the former, who is supposed to have lived about four and a half centuries prior to the Christian era, and from the origin of this doctrine to the present hour, it has ever had a formidable array of able and zealous advocates, and it comes down to the present generation inscribed with the sanction of time and experience, and of many and great names. And, basing our opinion upon medical history, we are justified in the conclusion that it has ever been, and continues to be, pretty universally accredited by the profession. And yet, strange to say, our own great countryman, the Father of American Medicine, the philanthropic and venerable Rush, was among those who opposed it. In his enumeration of the leading errors, as he supposed, of the learned and philosophic Sydenham, he mentions, among others, the following: Firstly, "A general belief in the salutary operations of nature in disease." Fourthly, "A belief in disease consisting in a vigorous effort of nature to throw off morbific matter." However, it should not be forgotten, that Dr. Rush was pre-eminently a *heroic* practitioner, and believed that "when the physician stepped into the sick room, nature should be politely asked to walk out." This, perhaps, was his greatest error, and may account, in part, for the magnitude of his bills of mortality. But the great Rush, and this his great error, are now numbered among the things that were. His distant successor, Prof. Wood, of Philadelphia, entertains, on this subject, sentiments diametrical to those of his renowned predecessor, as will be seen by consulting the following paragraph from his admirable work on the practice of medicine. He says: "It is a question of great importance how far it may be proper for the physician to interfere in the management of diseases. There is no doubt that too much may be and often has been done, and that as much evil may accrue from this course as from doing too little. The young practitioner should be strongly impressed with the truth, that in the majority of cases disease will end in recovery whether under treatment or not. He is not to suppose that every instance of recovery under his management is a cure. The prevalence of empiricism is in a considerable degree ascribable to this popular error, that a favorable termination of disease is always owing to the means employed. Patients often survive improper and even injurious treatment, and believing themselves cured, naturally acquire confidence in the practitioner or in the supposed remedy. Medical men may do much towards obviating the evil by imbueing the popular mind with accurate notions in this respect. But they should especially guard themselves against a participation in the error. An impression that medicines are essential or important in all cases, will often lead to their unnecessary and even to their injurious employment. It cannot be doubted that diseases which, if undisturbed, would have spontaneously terminated in health, have often received an unfavorable turn from officious interference. But it must not be inferred from what has been said, that the intervention of the physician is useless. On the contrary, even in the cases which would end favorably if trusted to nature alone, he may often do much good by shortening the duration of the complaint, alleviating the sufferings of the patient, and preventing inconvenient if not dangerous sequælæ. In many cases his aid is essential to the preservation of life. While, therefore, we guard ourselves against the evils of an overwhelming confidence in the efficacy of therapeutical measures, we should equally avoid the no less injurious influence of utter scepticism. In doubtful cases, where both reason and experience fail us, the best rule is to adopt the *expectant plan*, that is, to do little or nothing which can strongly impress the system, and await further developments, trusting in the meantime to nature." Upon this paragraph we have no comment to offer, except that it contains the words of "truth and soberness," and that we know not which most to admire, the candor of its author or the correctness of his views.

It has been said by a modern physiologist, that "life is the aggregate of those functions by which death is resisted," and we presume that the intelligent, and especially the medical philosopher, will admit that these functions, when in harmonious action, are capable, to a certain extent, of resisting the insalubrious influences of the surrounding agents common to our physical existence, otherwise man would be infinitely more short-lived than he is at present; life would truly be but a span, and he would only be born to pass, even more speedily than now, into physiological, physical and chemical dissolution. So long as it is admitted that disease may invade the system and afterwards become expelled without artificial or medical aid, so long are we authorized to rely largely upon the efforts of nature in the healing art, and to look to the *vis medicatrix naturæ*, not only as the rear but the van-guard of therapeutics, and as the sick man's best friend and anchor of hope in the hour of disease.

Modern authority, on this subject, is as cogent in Europe as in America. The celebrated Dr. Thomas Watson, of London, in his lectures upon the treatment of continued fever, says: "Continued fever, like other disorders which run a definite course, and have no direct or necessary operation in spoiling the structure of vital organs, has a strong natural tendency to terminate in health. We see this tendency when the disease is left entirely to itself, and it equally exists when remedies are employed to regulate its course or to abbreviate its duration. No one can doubt, who has had much experience in fever, that this tendency is sometimes thwarted by the *nimia cura medici*, and that patients get well in *spite* of the well meant but mischievous interference of the doctor. This tendency to recovery is a constant source, therefore, of fallacy in our observation upon the behavior of this disease under different plans of treatment, and upon the effects and utility of remedies. It leads us too often into danger of ascribing to drugs what is really due to the workings of nature, of confounding antecedents and sequences with causes and effects, of counting recoveries as cures. There are two things to be considered in the treatment of diseases; first, that we do the patient good; second, that *at least* we do him *no harm*." In all these exanthemeta, he must be reckond the safest and the best practitioner who knows when to abstain from acting as well as when to act, in other words, who has learned when and to what extent the case may be left to the *salutary processes of nature.*

However, there is an opposite error to that of mischievous activity, the tendency to recover, which manifests itself under different modes of treatment, and even in spite of opposite modes, has induced in some minds a degree of scepticism as to the utility of any remedies, that may easily be carried too far. It does not follow, because the majority of patients under continued fever would at length emerge into health, although no remedial measures were employed, that the disease ought therefore to be abandoned to what Cullen calls the "*vis medicatrix naturæ.* It is not quite correct to say with the older pathologists, that the whole disorder is merely an effort of nature to throw off something noxious to the system, and therefore is not to be intertered with. The true view of the matter, I apprehend to be, that which a toxicologist might take. The disease is produced by a poison, of which the injurious impression upon the animal economy at length passes off of itself, in the same manner, only more slowly, as the influence of a dose of opium will spontaneously pass away. But, during the natural course of the fever, as in many other cases of poisoning, morbid processes are apt to be set up, which, if suffered to proceed unchecked, would inflict irreparable injury upon important organs, and which are fairly within the scope of remedial management. Our object must be, when the fever is once established, to conduct it to a favorable close to "obviate the tendency to death." Upon this point, I agree most entirely with Pitcairn, who, being asked what he thought of a certain treatise on fever, declared: "I do not like fever curers—you may *guide* a fever, you cannot cure it. What would you think of a pilot who attempted to quell a storm? Either position is equally absurd. In the storm you steer the ship as well as you can, and in a fever you can only employ patience and judicious measures to meet the difficulties of the case."

No practitioner who has contended extensively with the epidemic (we allude to typhoid fever) that has by gradual but constant strides passed over our entire country of late, can gainsay the reasonableness, if not the correctness, of the preceding views of these distinguished teachers of medicine. They are founded upon reason and experience, free from ultraism, and are characterized by the wise maxim, "*In medio tutissimus ibis.*" These views, as already intimated, are not intended so much for the unbeliever as for the believer. We might enlarge almost *ad infinitum*, with authority, in support of this doctrine, but we deem it unnecessary, for to us it seems self-evident, and needs no argument in behalf of its establishment or maintenance. We might say much in regard to its connection with the etiology, semiology, diagnosis, and Therapeutic indications in special diseases, but lest our article should be protracted to an undue extent, we forbear further to enlarge. We are opposed to exclusiveness—no disciple of Broussais, no advocate of *masterly inactivity* in medicine—but impressed as we were with the important bearings of this great truth in medical philosophy, in the incipiency of our professional career, we have ever since made it the rule of our action, the index of all our therapeutic exertions, and after long observation upon its teachings, we find no sufficient reason to abandon our first convictions. On the contrary, we are more and more confirmed in the opinion, that it is our chief reliance for the removal of diseases, but certainly not to the exclusion of medicines, &c., as auxiliaries.

Intimately connected with the conservative power of nature, is the doctrine of *antistasis*, or counteraction—that is, the subsidence or removal of one form of morbid action by the occurrence of another peculiarity of morbid action, has usually been termed the *conversion* of diseases, or the cure of diseases by conversion. On this very interesting subject, and one as yet not fully understood, Walter Cooper Dendy, Esq., Surgeon to the Royal Infirmary for Children, in London, writes: "If we analyze the physiology of the living world, we shall find it displaying a series of antagonisms. The entire process of carrying on vitality, indeed, may be termed the resistance of one thing, to the influence or agency of another. In pathology it is essentially so. We daily see one disorder disappearing as another is developed or established." Hence, in the operation of all remedial agents, whether their action be "*similia similibus curanter*," or "*contraria contrariis opponenda*," the principle is the same. Thus we have poisons and antidotes, diseases and remedies, the former injurious and the latter palliative, or curative, and it is possible that every remedy owes its virtues, in a great degree, to the production of some abnormal influence less pernicious, however, and more transient in its effects than the one for the removal of which it is employed, and, theretore *antistasis* is regarded as an antagonistic force, vicarious action, &c., at the same time prophylactic or therapeutic in its tendencies and results.

Philosophers, long since, arrived at the conclusion, "that all *action* is followed by *re-action* equal to, but different from the primary action." And reasoning analogically from these premises, Dr. John Conolly, of the University of London, observes, that "New directions given to certain portions of the blood, together, probably, with a transference of morbid actions in the nerves, may cause not only the conversion of gout to mania already mentioned, but of erysipelas of the scalp to phrenites, or of erysipelas of the trunk to sub-inflammation of the intestinal coat with diarrhœa. The sudden repulsion of some of the disorders of the scalp in children as, of porrigo, is not unfrequently followed by meningitis. The sudden recession of the cutaneous efflorescence and inflammation in rubeola scarlatina, or variola, is commonly followed by inflammation of the lungs, or the bowels, or the brain."

We are therefore led to infer that the modes adopted by nature for the preservation of health and the expulsion of disease, are mainly (to use the language of Mr. Dendy) the processes of elimination and vicarious action, or antistasis. The former prophylactic, and the latter curative.

